# Edema-like marrow signal intensity: a narrative review with a pictorial essay

**DOI:** 10.1007/s00256-020-03632-4

**Published:** 2020-10-07

**Authors:** Davide Maraghelli, Maria Luisa Brandi, Marco Matucci Cerinic, Anna Julie Peired, Stefano Colagrande

**Affiliations:** 1 Department of Experimental and Clinical Biomedical Sciences, Radiodiagnostic Unit n. 2, University of Florence - Azienda Ospedaliero-Universitaria Careggi, Largo Brambilla 3, Florence, 50134 Italy; 2 Department of Experimental and Clinical Medicine, Unit of Bone and Mineral Diseases, University of Florence - Azienda Ospedaliero- Universitaria Careggi, Largo Brambilla 3, Florence, 50134 Italy; 3 Department of Experimental and Clinical Biomedical Sciences Division of Rheumatology, University of Florence - Azienda Ospedaliero-Universitaria Careggi, Largo Brambilla 3, Florence, 50134 Italy

**Keywords:** Edema-like marrow signal intensity, MRI, Rheumatoid arthritis, SIFK, AVN

## Abstract

The term edema-like marrow signal intensity (ELMSI) represents a general term describing an area of abnormal signal intensity at MRI. Its appearance includes absence of clear margins and the possibility of exceeding well-defined anatomical borders (for example, physeal scars). We can define “ELMSI with unknown cause” an entity where the characteristic MR appearance is associated with the absence of specific signs of an underlying condition. However, it is more often an important finding indicating the presence of an underlying disease, and we describe this case as “ELMSI with known cause.” It presents a dynamic behavior and its evolution can largely vary. It initially corresponds to an acute inflammatory response with edema, before being variably replaced by more permanent marrow remodeling changes such as fibrosis or myxomatous connective tissue that can occur over time. It is important to study ELMSI variations over time in order to evaluate the activity state and therapeutic response of an inflammatory chronic joint disease, the resolution of a trauma, and the severity of an osteoarthritis. We propose a narrative review of the literature dealing with various subjects about this challenging topic that is imaging, temporal evolution, etiology, differential diagnoses, and possible organization, together with a pictorial essay.

## Introduction

The term edema-like marrow signal intensity (ELMSI) represents a general term describing an area of abnormal signal intensity (SI) at MRI [1]. Currently, the term “ELMSI” replaces the old term “bone marrow edema,” which more properly represents a histopathological diagnosis, with the finding of an eosinophilic bone marrow extracellular fluid and swollen fat cells [1].

These areas of abnormal SI appear in very different conditions and are caused by various physiological and pathological processes. This SI alteration is usually caused by a pathologically detectable edema but can be also induced by medullary necrosis or blood products collection [1].

In fact, while MRI is well able to evaluate the evolution of heme-catabolic products, it does not easily distinguish cell swelling from the simple accumulation of extracellular fluids, or inflammation from fresh granulation tissue. So, what we define ELMSI is a non-specific but important finding usually indicating the presence of an underlying disease that can be found in many different pathological conditions (“ELMSI with known etiology”). Conversely, when it is found at MRI as an isolated finding without obvious cause, we can define it as “ELMSI with unknown etiology” [2, 3].

Based on this assumption, ELMSI has different implications in diverse diseases. Our aim was to highlight these variances and to propose a narrative review of the literature together with a pictorial essay trying to enhance the dialogue between clinicians and radiologists.

## Imaging

### Clinical standard

MRI is ideal for localizing the increased extracellular water of ELMSI and for the differential diagnosis of the associated disease [4, 5]. ELMSI appearance includes the absence of clear margins and the possibility of exceeding well-defined anatomical borders (for example, physeal scars) [4, 6].

It shows a low SI on T1-weighted (T1w) imaging and a high SI on T2-weighted (T2w, in particular if fat-suppressed) and on short T1 inversion recovery (STIR, another sequence adopted to saturate the signal of fat tissues) imaging [7]. Usually, after contrast agent administration [6, 8, 9], the enhancement is intense, and the SI even higher than on fat-suppressed and STIR acquisitions [9]. ELMSI develops from the periphery of a space-occupying lesion and it can be very complicated to differentiate it from the mass [10]. A further problem is given by the differentiation of ELMSI related to a malignancy compared to one related to a benign form: in recent years, this problem has been partially solved thanks to the adoption of diffusion-weighted imaging (DwI) and chemical shift-weighted imaging [11–13]. In fact, DwI with low *b* value allows to distinguish ELMSI caused by a vertebral fracture (trace, isointense vs.. normal marrow; apparent diffusion coefficient (ADC), high value) from that caused by a metastatic tumor infiltration of the spine (trace, hyperintense vs. normal marrow; ADC, low value) [14]. The enhancement pattern can help predict the evolution and the histopathological characteristics of ELMSI: a study differentiated ELMSI patterns in edema-like lesions, with a complete homogenization after contrast agent intravenous administration, and necrosis-like lesions, which showed incomplete and inhomogeneous enhancement [15]. At histopathological level, edema-like lesions were sustained by edema with accumulation of eosinophilic extracellular fluid into trabecular spaces at the periphery of the lesion; conversely, necrosis-like lesions were characterized by bone marrow fibrosis with necrosis areas, prevalently central.

### Research activity

STIR and T2w fat-suppressed images are very sensitive to detect ELMSI, but permit only a visual assessment [16], while DwI allows both qualitative and quantitative evaluations, which are helpful to better define the amount of cellular crowding or to give an estimate of the inflammatory degree [17]. The possibility of the latter assessment could be very helpful, especially in diseases as rheumatoid arthritis or ankylosing spondylitis, where patients’ stratification it very important for the individualized therapy and to predict disease progression. Even if to date there are no validated protocols for a universally recognized and confident ELMSI quantification, there are some promising sequences, such as IDEAL (i.e., iterative decomposition of water and fat SI with echo asymmetry and least squares estimation), based on multipoint Dixon fat water separation principle. The rationale is to ensure a uniform separation of water and fat, while providing images with a high signal-to-noise ratio. In fact, an optimal fat suppression could help develop a semi-automatic method for a correct quantitative assessment of ELMSI [18].

Perfusion-weighted imaging (PwI) was adopted as well in the attempt to give a prognostic assessment of ELMSI and to explain the related phenomena. The rate of contrast agent elimination was lower in ELMSI areas than in the unaffected bone, thus suggesting a venous obstruction blocking the outflow [19]. A significant correlation between ELMSI area dimension and the rate of contrast agent elimination was found, in patients with knee osteoarthritis in which perfusion parameters were investigated on PwI-MRI [20]. These results suggest that venous stasis could cause an intraosseous hypertension and consequently reduced perfusion and hypoxia. This mechanism could be responsible, at least in part, not only for pain but also for osteocyte stimulation and consequently bone remodeling [19]. Perfusion studies could be helpful in the differential diagnosis of three conditions characterized by ELMSI in the acute phase, but with great differences in terms of treatment and clinical outcome: transient osteoporosis of the hip, avascular necrosis (AVN), and subchondral insufficiency fractures (SIF) of the femoral head. Transient bone osteoporosis of the hip is usually spontaneously self-limited, AVN represents an irreversible condition possibly leading to permanent joint failure, and SIF may either completely resolve or progress towards osteonecrosis. Either conventional MRI acquisition or DwI does not always allow to differentiate these conditions [21, 22], but significant differences are found in perfusion parameters: maximum enhancement showed higher values in the transient osteoporosis and SIF, than in the AVN group. These higher values are maybe caused by reperfusion phenomena [23] that characterize transient osteoporosis and SIF, while the lower values in AVN are probably due to inflammatory processes, fibrocystic repair [24], and granulation tissue adjacent to the necrotic marrow [25]. Even if the co-presence of transient osteoporosis and SIF is not a univocal pattern [26], the differences in PwI reported above confirm that these conditions can be different spectra of the same disease [27].

Is it possible to detect a pattern equivalent to ELMSI by other acquisition techniques? While this detection by single energy CT has always been arduous due to the overlaying trabecular structure, dual-energy (DE-CT) techniques resulted able to discriminate water and fat, allowing the recognition of CT-pattern with value comparable to ELMSI. Moreover, virtual non-calcium DE-CT can visualize the marrow alterations in osteoporotic patients with acute vertebral fractures, helping to differentiate the older fractures from the recent ones [28] and DE cone-beam CT allows the detection of bone marrow edema and quantification of water and fat content in extremities [29].

## ELMSI as physiological finding

ELMSI is not always a pathological finding and may be observed in healthy children, where it is generally a temporary, self-limiting condition, particularly in the knee. Here, a focal peripheral edema at the growth plate is usually detectable, sometimes associated with pain. This is attributed to early stages of growth plate closure. A local ELMSI can be found in children during rapid growth at the spine too, as a transient finding decreasing over time, not related to physical activity. Other common frequent sites are the epi- and metaphysis of the distal femur, proximal tibia/fibula [30, 31], and at carpal bones/wrists [32, 33]. In these settings, ELMSI does not require invasive diagnostic procedures or imaging follow-up. Probable mechanisms include formation of metaplastic bone-neo-fibrocartilage, diminution in bone elasticity [31], local vascular alterations, and bone microtraumas [34].

ELMSI of the sacroiliac joints is another physiological condition and a very common finding in women screened for lower back pain. These marrow signal alterations have a predilection for the lower part of sacroiliac joint where stronger shear forces act, due to the increased weight carried in the anterior pelvis and the lordotic posture assumed by the mother. On the other hand, ELMSI of these joints represents a hallmark of axial spondyloarthropathies and the radiologist must not make the mistake of diagnosing these rheumatological diseases in peripartum women. The problem of misdiagnosis is real, considering that a significant proportion of these women with a physiologic ELMSI in the early period after labor fulfill the criteria of the Assessment of SpondyloArthritis International Society (ASAS) of a positive MRI for sacroiliitis, but these findings (and scores) decrease over time in the majority of the cases. For this reason, when an axial spondyloarthropathy is suspected, it is reasonable to wait at least 6 months to perform an MRI in postpartum women, and, if positive, repeat the exam after 12 months. The evolution of this physiological condition towards a true spondyloarthropathy seems coincidental, since the prevalence of peripartum women with ELMSI of the sacroiliac joints is not far greater than would be expected in a young patient population (< 4%) [35, 36].

Also, the phenomenon of conversion of the red marrow into yellow one is a physiological process and should not to be confused with the related ELMSI phenomena. Red and yellow bone marrows have different chemical composition. The first contains 40% fat, 40% water, and 20% protein, while the second one has 80% fat, 15% water, and 5% protein. The most sensitive sequences used for imaging of bone marrow changes are the GE in/opposed phase, when the H protons linked to water and fat are in the same voxel, and fat sat (STIR/SPIR/SPAIR) when the H protons linked to water and fat are in adjacent but different voxels. This is important to exclude the possible areas of ELMSI, which has a proportion of water protons higher than that of red marrow, and so a higher SI in T2w acquisition fat sat. The same acquisitions are important in the study of the reversal process too, i.e., bone marrow reconversion (from yellow to red marrow). This is a physiological response to increased hematopoietic needs of the body, including non-medical conditions, as can happen in “heavy smokers” or persons doing sports with a large oxygen need, and medical conditions, such as obesity (and related respiratory disorders), diabetes, anemia, and patients treated with hematopoietic growth factors [37].

## ELMSI temporal evolution

ELMSI presents a dynamic behavior [38, 39]: it goes through remodeling processes and its within-time evolution could largely vary at MRI study [40]. In the acute phase of an osteitis, ELMSI is mainly supported by edema but, in the subsequent phases of inflammation, bone marrow will undergo a remodeling process and ELMSI causative factors can be fibrosis or myxomatous connective tissue. For this reason, the medullary SI of the initial phases of inflammation (high water content) will be very different from the SI of the late phases (low water content) since ELMSI itself could vary in water content and size [40].

**Fig. 1:**
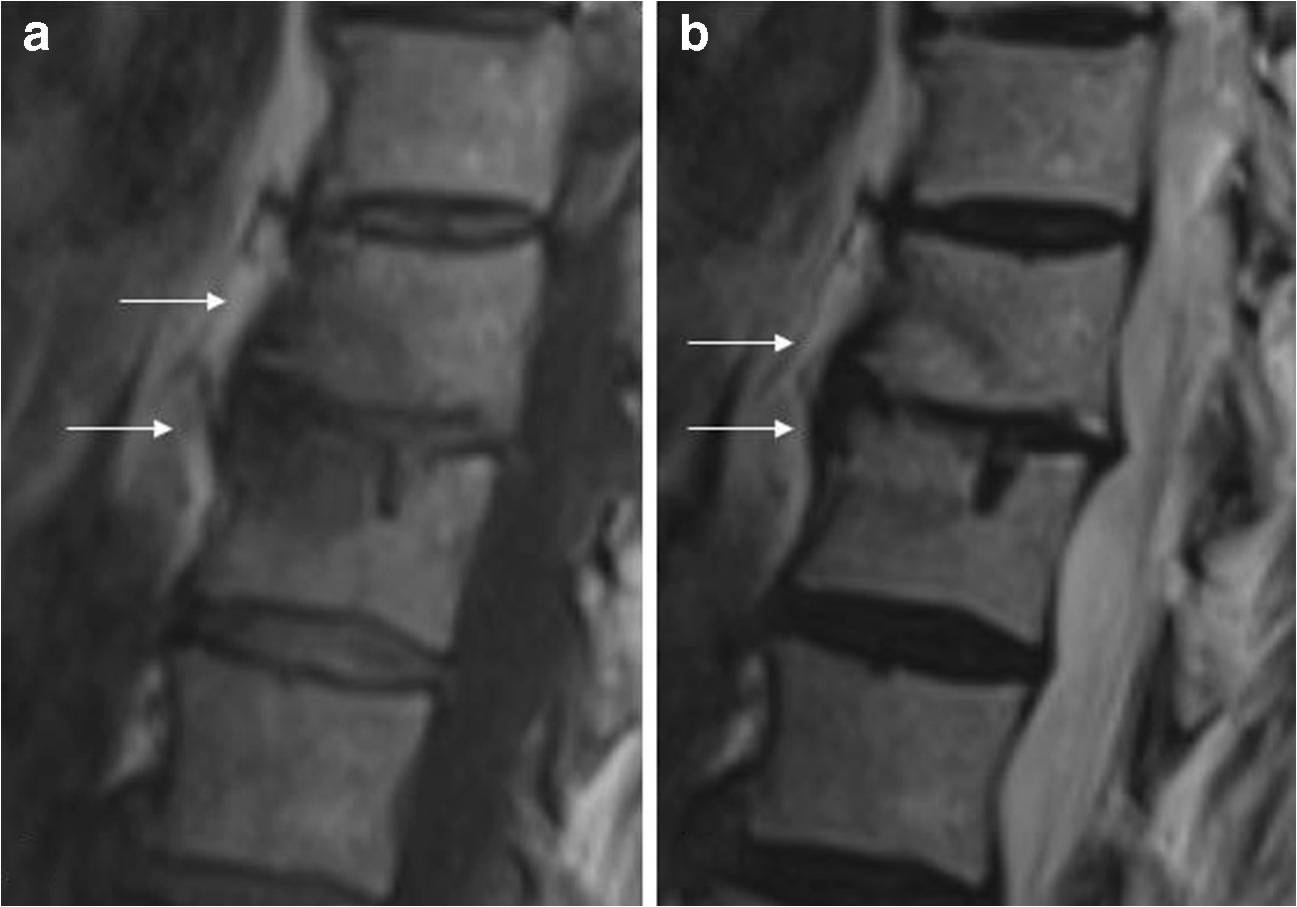
**a**, **b** Degenerative. Modic changes type I in a 78-year-old woman with back pain. MRI shows the SI alterations in D11 and D12 endplates (*arrows*), that appear hypointense on T1w (a) and hyperintense on T2w (b) imaging. These alterations correspond to vertebral body edema and hypervascularity

**Fig. 2:**
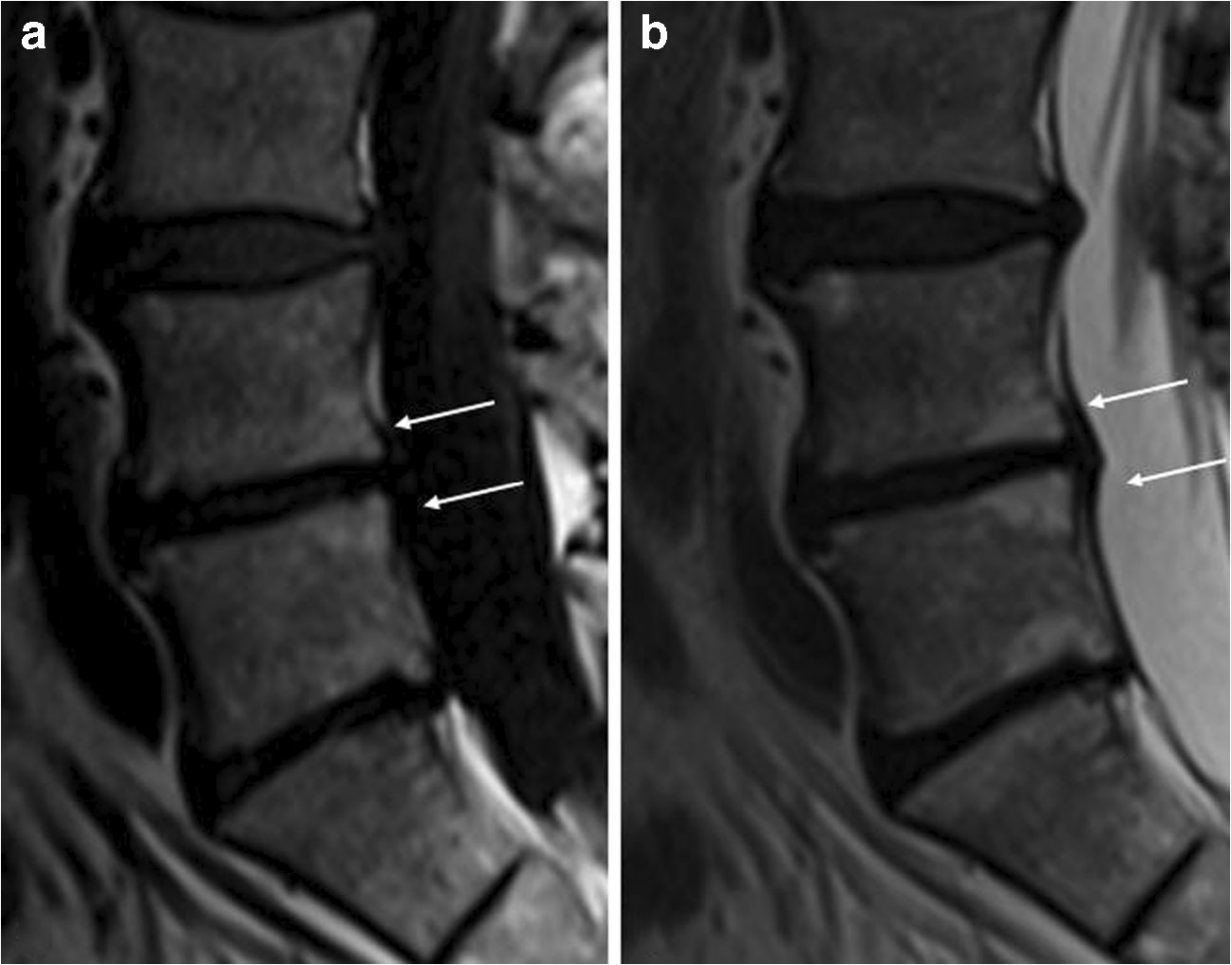
**a**, **b** Degenerative. Modic changes type II in a 53-year-old man. The hyperintense SI on T1w imaging of two lumbar bordering endplates (*arrows* in a) and hyperintense SI on T2w imaging (*arrows* in b) reflects fatty replacements of the red bone marrow, characterizing Modic changes type II

**Fig. 3:**
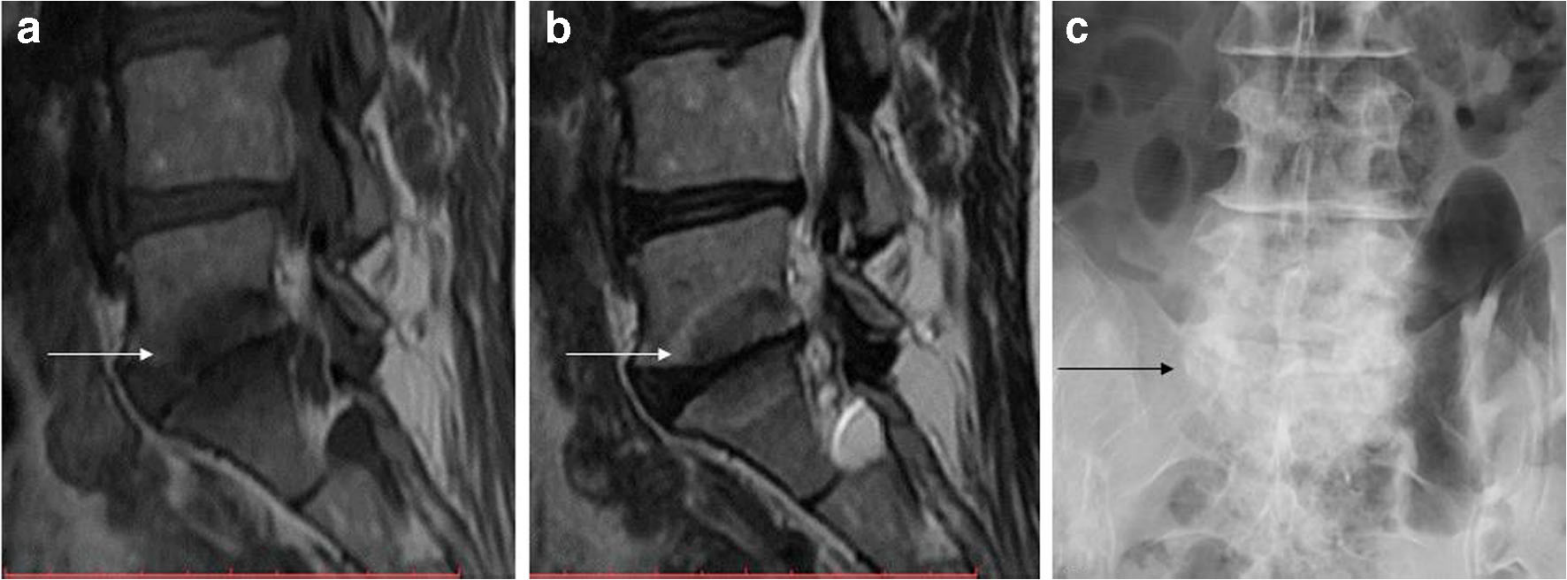
**a**, **b**, **c** Degenerative. Modic changes type III in a 53-year-old woman. The hypointense SI areas (*arrows*) both on T1w (A) and T2w (B) imaging reflect a subchondral bone sclerosis. The lumbosacral spine x-ray of the same patient (C) allows to better observe the bone sclerosis (*arrow*) of the caudal lumbar spine (courtesy of Giacomo Aringhieri, MD, University of Pisa School of Medicine, Italy)

ELMSI can undergo dimensional and SI fluctuations over time, especially depending on the activity/inactivity phase of the underlying disease, but these variations are not always associated with clinical changes. In osteoarthritis, ELMSI is a frequent finding, often fluctuating over a long-time period but the ELMSI dimensional reduction is not related with a decrease in the pain felt by the patient, valued via Western Ontario and McMaster Universities Arthritis Index (WOMAC) questionnaires [41]. The correlation between ELMSI evolution and the pain felt appears stronger in patients with transient knee osteoporosis [42], where a reduction in size of the edema is related to a reduction in the activity pain [42]. A classic example of such dynamic behavior of ELMSI are the so-called Modic changes, vertebral body endplate SI alterations often detectable in the MRI study of degenerative disk disease and spondylosis. These findings were first reported by de Roos [43] while Modic [44] gave the classification of these SI changes, as follows:

- Type I (SI low in T1w, high in T2w) corresponds to body edema and hypervascularity (Fig. 1)
- Type II (SI high in T1w, high in T2w) is a fatty infiltration of the bone marrow (Fig. 2).
- Type III (Si low SI in T1w, low in T2w) consists of subchondral sclerosis [45] and is rarer (Fig. 3).

The clinical importance of Modic changes is uncertain but it was hypothesized that Modic I alterations are related to the patient’s low back pain [46]. In fact, Modic I changes are associated with endplate neovascularity that would trigger vertebral nociceptors located precisely at this level (like those in the annulus fibrosis and posterior longitudinal ligaments).

## Histopathological findings in ELMSI

The MRI pattern of ELMSI is caused by various occurrences related to a replacement of normal fatty bone marrow by a more water-rich tissue [47]. This change was often assumed to be due to a real local edema, but only few studies confirm this hypothesis [48]. On the contrary, as we have seen above, in most histological samples, it was found prevalently lymphocytic infiltrates, fibrosis, improved vascularization, and reduced mineralized bone. These findings, however, should be considered with caution due to inevitable limitations related to the time passed between ELMSI appearance and histological examination. To summarize the occurrences found in literatures, the histopathological meaning of ELMSI could be different in distinct clinical entities, as follows:

1. A simple real edema and a reduced bone mineralization in transient osteoporosis; these are usually self-limiting and recover in a variable time, without sequelae in most cases [49].
2. A leakage of interstitial fluid and hemorrhage into marrow spaces [8], as consequence of acute or chronic repeated traumas inducing marrow trabeculae disruption.
3. An inflammatory infiltrate in rheumatoid arthritis (RA); in this case, the edema can be mediated by local cytokines related to trauma and hypoxia. The local release of pro-inflammatory cytokines in the presence of an inflammatory infiltrate has been documented in inflammatory arthritis such as RA, where bone marrow fat is replaced by aggregates of lymphocytes and blood vessels [47].
4. A fibrosis and bone marrow necrosis in advanced osteoarthritis.
5. A vascular alteration as an increased marrow blood flow (hyperemic) or an impaired vascular drainage (congestive) [8]; both conditions can cause an increased intraosseous pressure which can lead to a diminished perfusion and hypoxia [19].

## Etiology and organization

**Fig. 4:**
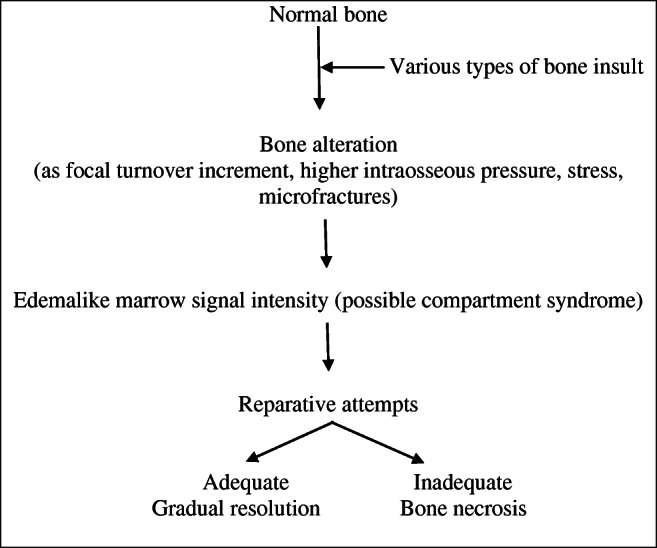
Bone insult cascade. Adapted from references [50, 51]

There is still a poor understanding of the pathological mechanisms underlying ELMSI. The most recognized mechanism, the “bone insult cascade,” which determines its onset and possible evolution towards resolution or osteonecrosis is summarized (Fig. 4) and an adapted classification according to putative risk factors is reported (Table 1). For a radiologist, the earliest moment linked to ELMSI is obviously its recognition; consequently, it is important to figure out the pathological process by trying to subcategorize EMLSI as “with known cause” and “with unknown cause.” The latter category should only be diagnosed once every possible underlying cause has been excluded. The various etiological subcategories will be discussed in detail in the following sections, according to the best-known classification [54].

**Table 1:** Classification of ELMSI with known etiology, adapted from references [52, 53]

Type	Etiology	Main imaging features
Trauma	Direct or indirect damage, fracture, CRPS	- The stress injury-induced ELMSI is often subchondral and wedge-shaped with the base located at the site of greatest stress - The bone bruise/contusion appears as an area of heterogeneous ELMSI - ELMSI is larger if due to compressive rather than traction forces - If a fracture is present, ELMSI is accompanied by the hypointense line in all sequences - The CRPS-induced ELMSI typically shows a patchy pattern
Trauma/degenerative	SIF	- Ill-defined ELMSI arising from the subchondral region with a fracture line in the subarticular marrow - Ischemic low T2w-SI subchondral area subjacent to the collapsed bone plate - Fluid-filled cleft underlying the collapsed bone plate
Degenerative	Osteoarthritis	- Joint-sited ELMSI due to high metabolic activity, extracellular matrix turnover/angiogenesis, and bone formation - ELMSI is frequently associated with geodes, reactive synovitis, joint effusion and loss of joint space - More evident ELMSI when greater cartilage loss or abnormalities occur
Degenerative/inflammatory	Modic changes	- Type I (low SI in T1w and high SI in T2w) corresponds to vertebral body edema and hypervascularity - Type II (high SI in T1w and T2w) reflects fatty replacements of the red BM - Type III (low SI in T1w and T2w) consists of subchondral bone sclerosis
Inflammatory	Inflammatory arthritis, enthesitis	- ELMSI is caused by the replacement of the medullary fat with inflammatory cells with edema - Immature blood vessels containing high levels of VEGF can also contribute to ELMSI appearance - In RA patients, ELMSI is representative of bone inflammation, initially located in the bare areas - In these patients ELMSI and synovitis precede the appearance of bone erosion and joint space narrowing and - The progression of joint destruction is significantly greater in ELMSI positive rather than in negative joints
Vascular	Avascular necrosis (AVN)	- ELMSI is sited in the viable tissue and surrounds the area of necrotic marrow outlined by a low SI rim - This rim is often double-lined on T2w acquisitions: a high inner (granulation tissue) and an outer low SI band (sclerosis) - AVN-related ELMSI seems not to represent an early finding and could be secondary to a subchondral fracture
Infectious	Bone and articular infections	- ELMSI frequently surrounds a mass of infected tissue - Septic arthritis has a wide spectrum of imaging presentation and can be divided in: - Necrotic, caused by an ischemic mechanism - Exudative, induced by vascular congestion (DD with gelatinous transformation of bone marrow, due to protein loss)
Neoplastic	Benign lesions	- Benign lesions as osteoid osteoma and osteoblastoma show an important ELMSI - Usually ELMSI seems caused by trabecular destruction and local inflammation, but - Osteoid osteoma surrounding ELMSI seems due to tumor-associated inflammatory mediators (PGE_2_)
Neoplastic	Malignant lesions	- Generally, primary/secondary malignancy induces a minor quantity of ELM with respect to benign tumors but - Chondroblastoma and Langerhans’s cell histiocytosis are surrounded by an intense ELMSI -DwI can differentiate the neoplastic focus from the surrounding ELMSI
Iatrogenic	After surgery or RT, steroids or calcineurin inhibitors	- ELMSI RT-related is the most frequent - In this case usually presents fast onset and short duration (1-14-day time frame), however - Its presence, intensity and duration can vary significantly depending on treatment type/location
Metabolic	Hydroxyapatite deposition disease, CPPD, gout	- ELMSI is due to the inflammatory response at pathologic tendon insertion site - This reaction is due to hydroxyapatite or calcium pyrophosphate deposition - In gout can be caused by the presence of intraosseous tophi
Neurological	Charcot’s joints	- ELMSI is often associated with this disorder commonly affecting foot and ankle - ELMSI is an early sign of Charcot’s joint - MRI is sensitive to follow the course of the healing process and differentiate acute Charcot’s foot from acute osteomyelitis

### Edema-like marrow signal intensity with unknown cause

**Fig. 5:**
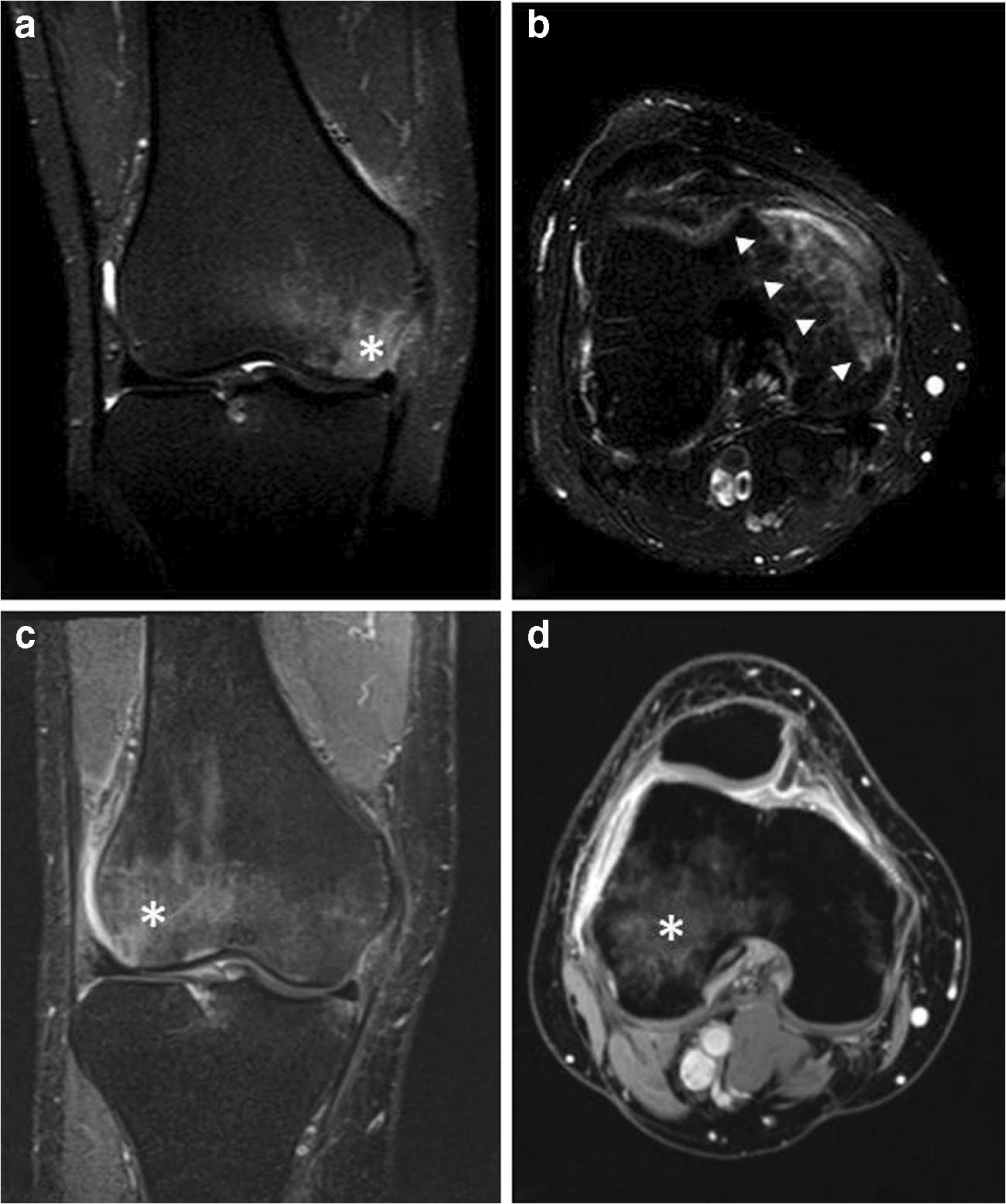
**a**, **b**, **c**, **d** Primary. A 53-year-old male patient with pain, swelling, and stiffness of the left knee. STIR imaging from the first MRI exam (a, b) highlights edema (A single asterisk (*) in a and arrowheads in b) in the medial condyle; degenerative phenomena affecting meniscuses, articular cartilage and ligaments are not present. STIR imaging performed after two months (c, d) shows that edema (*), from the medial compartment of the knee, has moved into the lateral compartment of the same knee, while all the other findings observed at the first examination remained unchanged. Every possible etiology has been excluded: this is an example of migratory primary ELMSI

This category can include all those diseases characterized by ELMSI as a main, often unique, sign and whose etiology is not defined, as “transient bone marrow edema syndrome,” “transient regional osteoporosis,” “transient regional bone marrow edema,” “migratory osteoporosis” [54]. In the past, depending on the medical specialist involved (rheumatologist, orthopedic, or radiologist), the use of different terms to specify the same condition (for example, “transient regional osteoporosis” or “transient regional bone marrow edema”), has led to some misunderstandings as to what exactly was meant [55]. Unfortunately, it is not possible to assert that it is always spontaneously resolving: the cases of ELMSI with unknown etiology are too few to reach this conclusion and there are some possible cases of transient edema syndrome that have progressed towards osteonecrosis [56]. On this background, we could define primary ELMSI as a usually self-limiting condition with unknown etiology, resolving without long-term sequelae and/or necessity of surgical treatment [54], which may migrate to involve another bone in a same region or a distant bone in the same or contralateral limb. As being understandable, only an “a posteriori diagnosis” is feasible [55] **(**Fig. 5). The best known of these disorders is the transient osteoporosis of the hip, a disorder most common in young men and women in the last trimester of pregnancy, predisposing them to fragility fractures. The SI alteration is usually located at the femoral head and may extend to the femoral neck and intertrochanteric region and is often accompanied by joint effusion. The delayed peak enhancement of edematous marrow is characteristic of this condition [57].

### Edema-like marrow signal intensity with known cause

#### Trauma

**Fig. 6:**
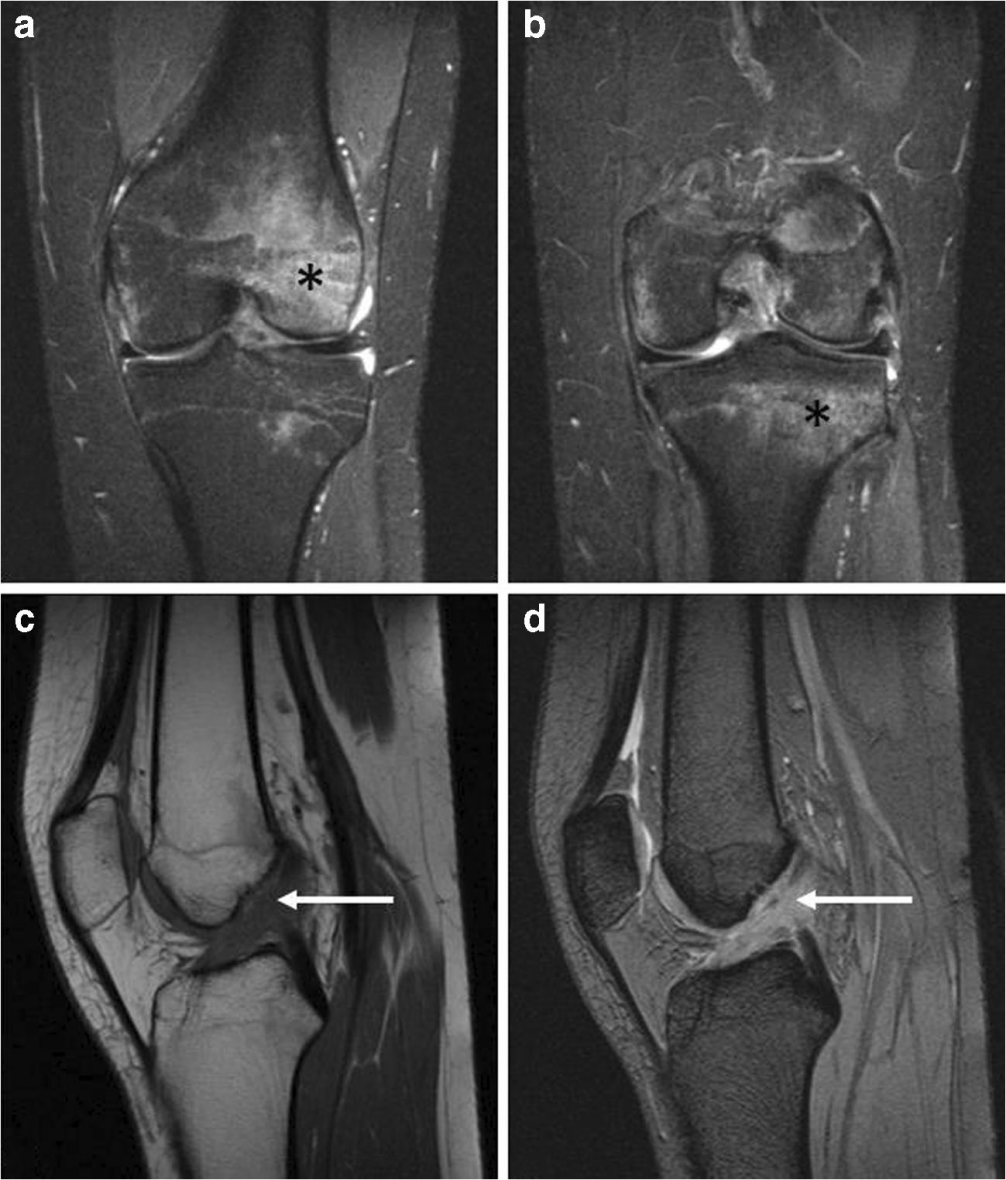
**a**, **b**, **c**, **d** Traumatic. A 17-year-old female with a traumatic ELMSI after a contusion of the right knee. STIR coronal imaging (a, b) demonstrates the presence of the hyperintense ELMSI (*) in the anterior side of the lateral femoral condyle (a) and in the lateral side of the tibial plateau (b). The presence of an abundant marrow signal alteration is often correlated with a ligament injury: in this case, the anterior cruciate ligament (ACL) is scarcely recognizable (*arrows*) both in the T1w (c) and T2w (d) sequences: the finding is compatible with a high-grade ACL injury

It represents one of the most important and frequent mechanisms in determining ELMSI. The traumatic mechanisms underlying its onset are manifold (biomechanical stress injury, bone bruise/contusion, fracture) and MRI can help in identifying them (Fig. 6). The stress injury–induced ELMSI is often subchondral and wedge-shaped, with the base located at the site of greatest stress or load; the bone bruise/contusion appears as an area of heterogeneous marrow alteration, larger if it is due to compressive forces rather than traction forces; in a fracture, it is accompanied by the presence of the hypointense line in all sequences [58]. Also, especially in the knee, ELMSI is statically linked to many ligament and meniscal injuries, so its presence can be helpful to focus attention on subtle but important abnormalities that might otherwise be missed, such as popliteo-fibular ligament damage and meniscal [59].

**Fig. 7:**
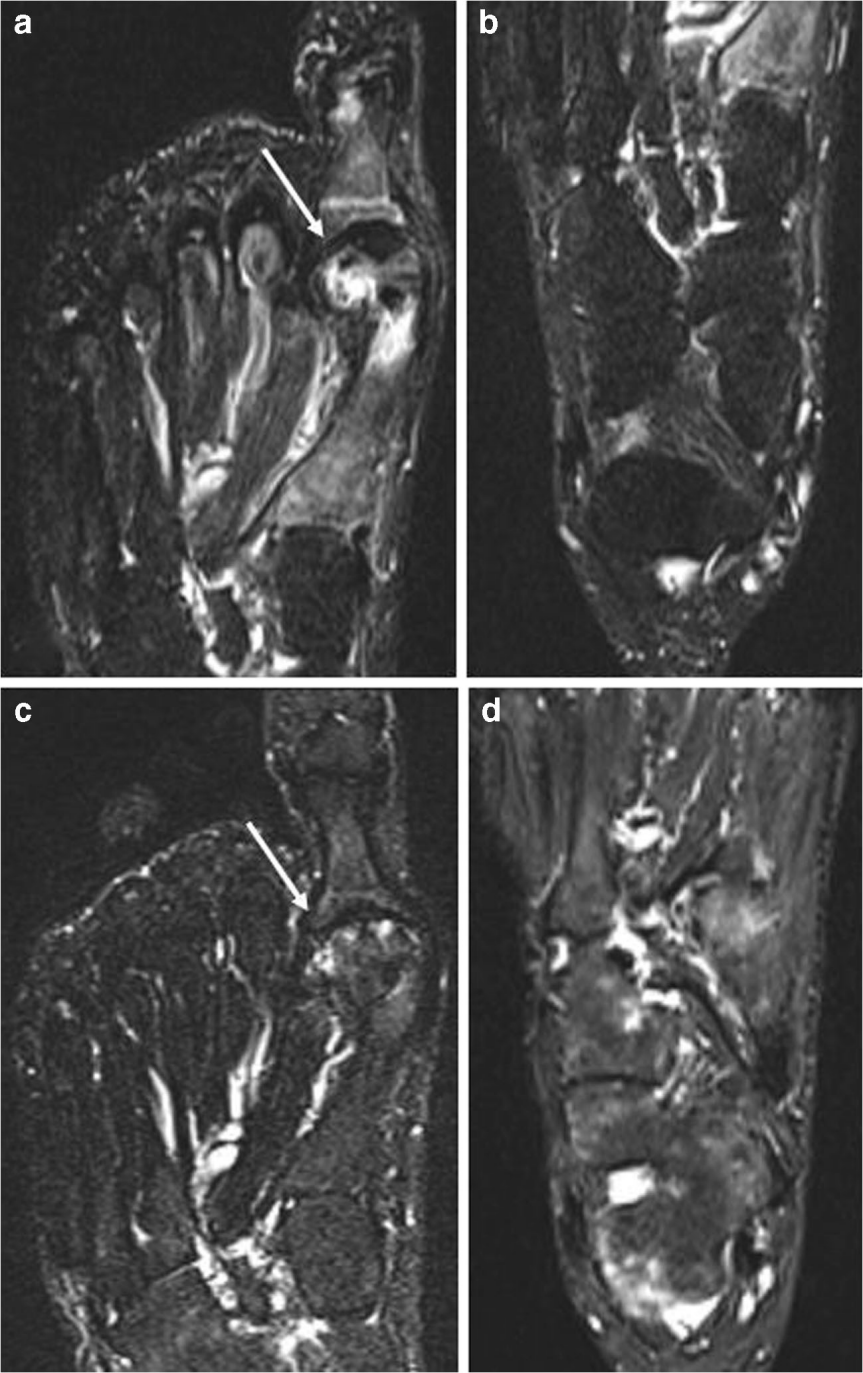
**a**, **b**, **c**, **d** Traumatic (CRPS) and iatrogenic. A 58-year-old woman that underwent surgery for left hallux valgus correction. All the figures are STIR images. Panels a and b come from the first exam, few weeks after surgery, where it is possible to observe an ELMSI involving the first metatarsal head (*arrow* in a) compatible with the post-surgery (iatrogenic) ELMSI; no ELMSI of the tarsal bones (b). After 6 months, the patient continued to feel pain in the left foot and MRI was repeated. MRI shows the almost total remission of the ELM in the surgical site (*arrow* in c), confirming the alteration was a simple post-surgery (iatrogenic) ELMSI, but its presence with “patchy” distribution in the cuneiform bones, in the cuboid, in the talus, in the calcaneus, associated with effusion in the tarsal and ankle joints (d), led to the diagnosis of CRPS

The etiology of ELMSI caused by subchondral insufficiency fractures of the knee (SIFK, which replaces the old term spontaneous osteonecrosis of the knee, SONK) can be considered, in part, traumatic as linked to these micro-fractures in the porotic bone. SIFK is at present known to be caused by insufficiency fractures, with fluid accumulation in the bone marrow, subsequent edema with focal ischemia, and eventual necrosis, however rarely observed [60]. SIFK are typically unilateral, most seen in 60-year-olds or older women [61] and are classically described as focal, superficial subchondral lesions, mainly affecting the medial femoral condyle [62]. A strong association between meniscal tears, particularly of the medial posterior root, and SIFK has been recently observed [60], probably due to the consequent increased contact pressures. MRI features of SIFK include an ill-defined ELMSI in the subchondral region, the hypointense rhyme of fracture with the collapse and separation of the bone plate [62–64], and a subchondral area of low SI on T2w located immediately subjacent to the bone plate. This area, representing local ischemia, is very important because if it is thicker than 4 mm or longer than 14 mm, the lesion may be irreversible and may evolve into irreparable epiphyseal collapse and articular destruction. For this reason, this area acts as the most important prognostic indicator of more rapidly progressive lesion [65, 66]. Unfortunately, ELMSI does not appear to have a prognostic role in prediction of poor clinical outcome because diffuse marrow changes can be observed in the early stages of irreversible osteonecrosis and in transient lesions as well. Furthermore, no correlation with the ELMSI extension has been observed [65, 67]. In the acute phase after pain onset, ELMSI increases both in intensity and extension and a small subchondral sclerosis could appear on T1w imaging, while radiography remains unremarkable. If the condition is recognized and properly treated, in the following weeks, MRI will show subtotal regression of marrow alterations and over the next few months the lesion will continue to decline, but changes could remain even after 2.5 years [68]. ELMSI from complex regional pain syndrome (CRPS) (Fig. 7) can be included in this category. CRPS can develop secondary to any trauma, and is characterized by a continuing (spontaneous and/or evoked) regional pain that is disproportionate in time or degree to the usual course of any known trauma or other lesion and is believed to be caused by damage to, or malfunction of, the autonomic nervous system. Typical MRI findings are patchy ELMSI and diffusely increased juxta-articular fluid [69].

#### Degenerative

The MRI presentation of osteoarthritis includes geodes, reactive synovitis, joint effusion, osteophytes, loss of joint space, and subchondral ELMSI. The edema is more frequent in those patients who have greater cartilage loss or abnormalities [58]. Symptoms of impaired function in osteoarthritis are influenced not only by joint synovitis and effusion, tendon, and ligament involvement but also by the presence of ELMSI, which represents regions of high metabolic activity with increased extracellular matrix turnover, angiogenesis, and bone formation [70]. Although a precise statistical correlation between its extension and pain has not been proved yet [41], multiple studies have observed this relationship. Ahedi [71] found that the presence of large hip ELMSI in osteoarthritis patients was associated with overall fourfold higher odds of hip pain and that an increase in hip ELMSI size over time was related to a progressive increase in pain. Furthermore, a high cartilage signal, reflecting deleterious changes in articular cartilage, may be asymptomatic because it is not directly related to pain, although it is strongly associated with the presence of large hip marrow alterations (most notably acetabular) [71]. Unfortunately, the studies correlating the extent of ELMSI and joint pain are too few and further studies are needed in the future to clarify a precise correlation. In this context, we should not forget the Modic changes, which can contribute to evaluate the spinal degenerative disease (Figs. 1, 2, 3).

#### Inflammatory

**Fig. 8:**
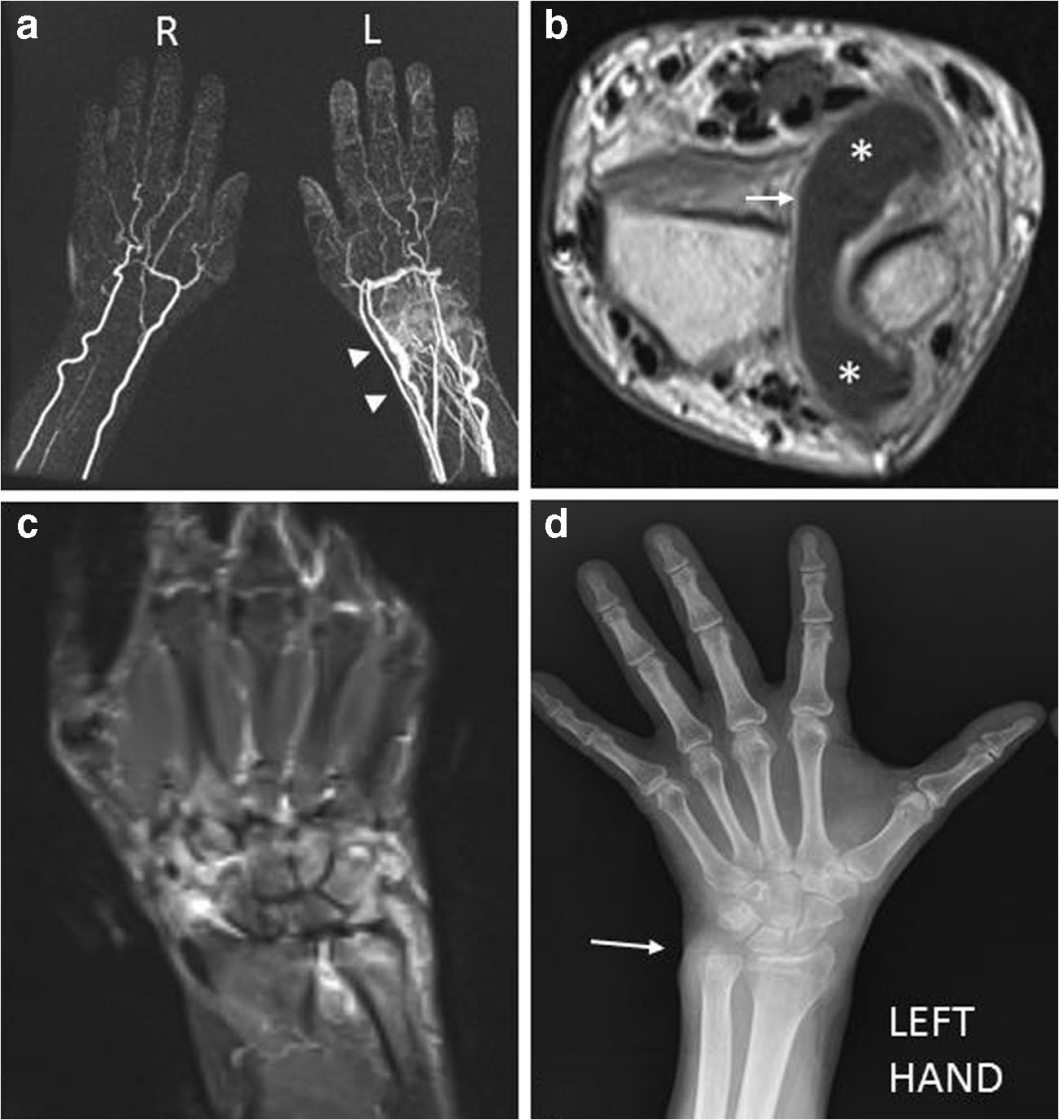
**a**, **b**, **c**, **d** Inflammatory. A 56-year-old rheumatoid arthritis (RA) male patient with pain and swelling in the left wrist. TWIST Angio 3D MIP imaging (a) clearly shows the inflammation with hypervascularization and edema (*arrowheads*) affecting the left wrist. Contrast-enhanced T1w imaging (b) demonstrates an abundant intra-articular effusion in the distal radioulnar joint (*) with thickening and enhancement of synovia (*arrow*), a condition referable to inflammation in the active phase. STIR coronal imaging (c) highlights the ELMSI affecting part of the ulna, lunate, triquetrum, hamate and capitate bones, and the effusion in radioscaphoid and radioulnar joints. Finally, the X-rays of the hand (d) taken at the same time shows a “soft-tissue sign” near the ulnar styloid process (*arrow*). but absence of erosions, while MRI is strongly positive for active inflammation [courtesy of Giovanni D'Elia, MD, Careggi University Hospital, Florence, Italy]

**Fig. 9:**
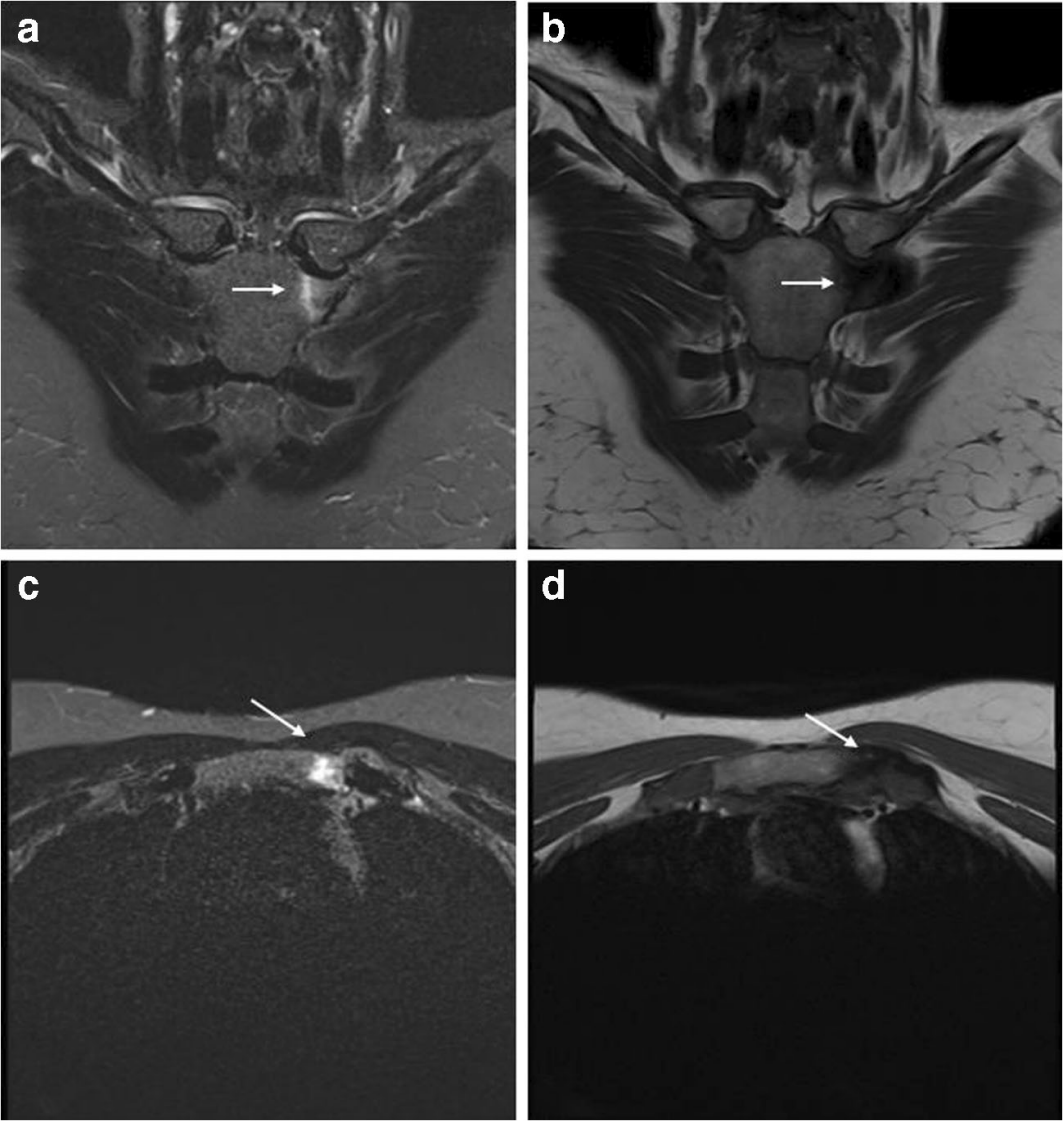
**a**, **b**, **c**, **d** Inflammatory. A 49-year-old woman with swelling and pain in the left sternoclavicular joint and history of psoriasis. MRI highlights the ELMSI of the joint (*arrows*), which is hyperintense in STIR (a–c) and hypointense in T1w (b–d) imaging. This is a very particular condition and can be referred to either an early SAPHO (synovitis-acne-pustulosis-hyperostosis-osteitis) syndrome, which is often connected with a special form of psoriasis (pustulosis palmoplantaris) and has an inflammatory involvement of the sternoclavicular joint, or a Tietze syndrome, a painful inflammation that can affect the costochondral, costosternal, or sternoclavicular joints

ELMSI can be observed in many musculoskeletal inflammatory pathologies as RA (Fig. 8): in this case, it is a product of bone inflammation, consisting in the replacement of the medullary fat with a cellular infiltration leading to an increase in the local water content and edema. According to one of the most accredited theories, ELMSI is initially located in the “bare areas,” regions of bone that are uncovered by cartilage, where the pannus, which is a hyperplastic synovium filled with inflammatory cells and dilated vessels, acts on the underlying marrow [58, 72]. ELMSI appears after a few weeks from occurrence of symptoms and correlates with elevated markers of acute inflammatory phase; furthermore, it is a reversible phenomenon and may become attenuated due to biological treatment [73]. As with RA, it also intervenes in the pathogenesis of other rheumatological conditions, such as inflammatory arthropathies in general and systemic diseases causing chronic inflammation and fibrosis [58]. In RA patients, ELMSI is a common finding, most frequently in the upper extremities but described in all possible locations of disease; it can be found already from the early stages of RA, but it could arise even later. American College of Rheumatology/European League Against Rheumatism (ACR/EULAR) guidelines suggest the use of ultrasound in early RA to assess the activity of the inflammation with respect to MRI, that is reserved for a small portion of clinically difficult cases and clinical studies. So, in these patients, the study of ELMSI seems less important than the findings visible with ultrasound [74]. Taking this into consideration, we must however recognize the information that ELMSI can provide once it has been observed in a RA patient: the detection of synovitis and ELMSI allows the aggressive forms of RA to be recognized earlier because bone erosion and joint space narrowing are invariably preceded by these phenomena. Moreover, the progress of joint destruction is significantly greater in ELMSI positive rather than in ELMSI-negative joints [75]. Many studies recognize the presence of ELMSI as the strongest individual predictor of bone erosions. Other factors such as the RA MRI score (RAMRIS) for synovitis, C-reactive protein, and anti-cyclic–citrullinated peptide status did not achieve a comparable predictive significance [76–83]. The risk of forming erosions is 6-fold higher in areas where ELMSI was noted earlier [73]. Moreover, if it was once thought that erosions only developed in joints where synovitis was and hampered in the joints where synovitis had been suppressed [84], McGonagle [85] has observed progression towards erosion formation in joints without clear MRI traits of synovitis. In these patients, it was only possible to observe a very slight thickening of the synovium but, above all, abundant marrow alterations. It is thought that synovitis does not directly cause erosions, but rather indirectly induces them through ELMSI: inflammation of bone marrow causes the migration of inflammatory mediators and cells into the joint cavity through enlarged channels within bone tissue, leading to synovitis and, in an indirect manner, to erosion formation. In this model, ELMSI would appear earlier than synovitis [73]. Furthermore, it may be a more sensitive and earlier indicator of the response to therapy than appearance of the synovium thickening [73]. In patients receiving anti-TNF-α therapy, there is a significant reduction in OMERACT (Outcome Measures in Rheumatology) scores both of synovitis and ELMSI with significantly less erosive progression at 1 year, but the reduction of the latter occurs earlier (1 month vs. 3 months) [73]. ELMSI is also the most important predictor of progression to criteria-positive RA in patients with undifferentiated arthritis, both independently and to a greater extent when combined with anti-cyclic–citrullinated peptide status or rheumatoid factor positivity [86]. In the earlier phases of the disease, it is usually located in the subchondral bone, well seen in the metacarpophalangeal or proximal interphalangeal joints. However, it cannot be detected in all patients with early RA: literature reports that ELM is present in 40% of cases of early RA (< 3 years’ duration) and in 70% of cases of established RA (> 3 years’ duration) [87]. The clinical distinction between spondyloarthropathies involving peripheral joints and RA is not so simple (for example, in psoriatic arthritis “sine psoriasis”) (Fig. 9) and traditional radiography is often not conclusive. Although clinical manifestations, serological and antibody biomarkers, radiography and ultrasound are sensitive and specific tools for differential diagnosis, MRI is useful for cases that remain uncertain. Its role in distinguishing the various arthritis types, it has been much debated over the years and various authors reported that MRI is not able to distinguish among these entities at the individual case level, although differences at the group level are evident [88]. Some findings may therefore be useful, such as a diffuse ELMSI adjacent to the enthesis, as well as florid inflammatory soft tissue changes at this site, which represents a clear sign of enthesitis, considered the hallmark of the peripheral forms of spondyloarthropathy. Another MRI finding of psoriatic arthritis is the ELMSI observed in the diaphysis of the phalanges at a considerable distance from the subchondral bone and the capsular joint entheses. Instead, in patients with RA, it usually occurs adjacent to cartilage in the subchondral bone and is less extensive than in patients with spondyloarthropathies [87].

#### Ischemic

**Fig. 10:**
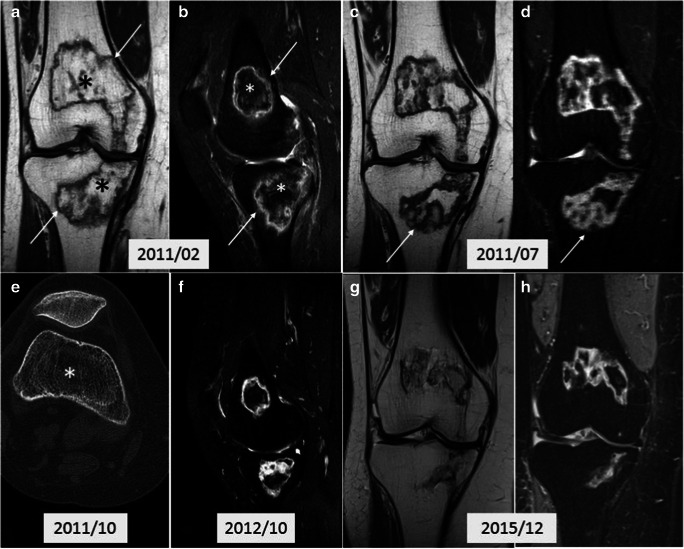
**a**, **b**, **c**, **d**, **e**, **f**, **g**, **h AVN**. A 53-year-old woman with pain during mobilization of the right knee; MRI has raised the suspicion of AVN (bone infarct) and we can note the evolution of the findings over 4 years. T1w (a) and proton density fat–suppressed (PD-FS, b) imaging show two areas of altered signal in the distal end of the femur and in the proximal end of the tibia with irregular morphology and inhomogeneous structure (*), with a peripheral rim, hypointense in T1w (*arrows*) and hyperintense in PD-FS (*arrows*) imaging corresponding to the reactive interface along the margin of infarct. Three months after the previous MRI (c, T1w and d, PD-FS), a further increase in the interface (*arrows*), a reduction in the ischemic area and signs of regeneration of the cancellous bone are observed. While the MRI findings are well evident, the CT performed in the same period (e) shows only a limited rarefaction (*) of the cancellous bone. One year later (f, T2w-FS) the infarct outcome area is still evident but smaller compared to previous examinations. Four years later (g, PD and h, STIR) the infarct zone appears less extensive and more shaded, but still present; the interface appears larger. ELMSI had not been detected for all 4 years and the patient progressively underwent an improvement in the clinical situation, with the disappearance of the pain. It is important to underline that ELMSI represents a fundamental indicator of a subchondral fracture and a progression towards epiphyseal collapse, which in this case did not occur

This category is represented by ELMSI caused by AVN, a generic disease that may have different predisposing conditions; it is more frequently found in younger patients than SIFK and is related to different predisposing conditions (sickle cell anemia, myeloproliferative disorders, alcohol, corticosteroids, and tobacco consumption) (Fig. 10). AVN patients experience pain (with a gradual onset) localized in the joint district affected and sometimes the pain involves multiple districts since AVN may interest multiple joints. In the knee, the femoral condyles are the most involved, but in about 20% of cases, the tibial plateau may also be affected [89]. In an early phase, AVN presents with a medullary necrotic area delineated by a hypointense sclerotic rim representing the border between necrotic and vital bone that surrounds the infarcted area without interruption. Often, in T2w sequences, an inner high SI band, given by the granulation tissue, and an outer low SI band, given by the bone sclerosis, are visible; these two bands form the so-called “double-line sign.” In recent years, many scientists have questioned the role of ELMSI within AVN disease. In AVN of the femoral head, several authors have observed that ELMSI does not represent an early finding, as it is a secondary to a subchondral fracture. ELMSI in AVN correlates highly to the subsequent collapse of the femoral head, resulting in clinical worsening of hip pain. The occurrence of a subchondral fracture in AVN defines stage 3 ARCO (Association for Research on Osseous Circulation) and makes conservative treatment of the disease impossible. For this reason, the detection of ELMSI in AVN of the femoral head must lead to a careful search for a fracture rim. If MRI fails to detect it, it is advisable to perform a CT which is more sensitive. If the fracture is confirmed, joint replacement or bone grafting is suggested. ELMSI in AVN of the femoral head may represent an inflammatory change in the reactive process during the progression of collapse of the femoral head and might be a sign for the deterioration of the disease [90].

#### Infectious

**Fig. 11:**
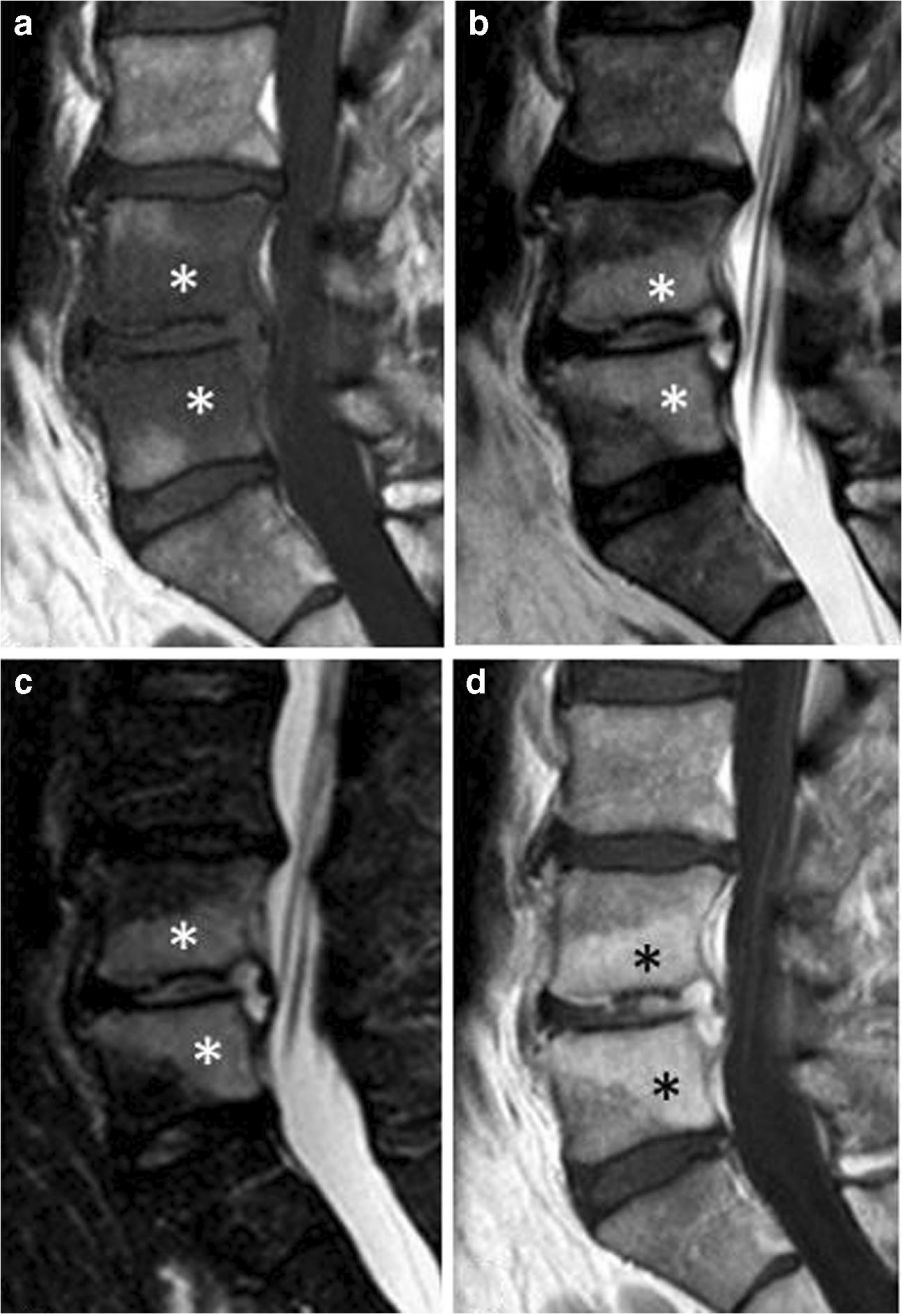
**a**, **b**, **c**, **d** Infectious. Spondylodiscitis involving L4–L5 in a 52-year-old man with fever and low back pain. MRI shows a large ELMSI (*) of the vertebral bodies, appearing hypointense in T1w imaging (a), hyperintense in T2w (b), STIR (c), and contrast-enhanced T1w imaging (d). The interposed disc is also involved in the inflammatory process, appearing hyperintense on T2w, STIR and contrast-enhanced T1w sequences

ELMSI can be associated with bone infections such as spondylodiscitis (Fig. 11) or osteomyelitis, and articular infections (infectious arthritis). Bone infections can be divided in exudative and necrotic forms. In the latter, ELMSI is caused by an ischemic mechanism, while the exudative form is induced by artero-capillary congestion, and, in this case, it is important to differentiate (via history and/or bone marrow aspiration) ELMSI from the gelatinous transformation of bone marrow, a typical sign in patients with severe malnutrition and protein loss. MRI commonly shows a mass of infected tissue surrounded by a variable volume of ELMSI. Septic arthritis has a wide spectrum of imaging presentation: sometimes it appears with a full panel of signs as ELMSI with concomitant changes in the cartilage, joint capsule, and soft tissues. However, it could appear also with only one sign and with small expressivity. Regardless of the imaging presentation, during treatment, clinical symptoms usually regress faster than the appearance of ELMSI on MRI, and it has been noted that MRI findings can be of assistance in determining when to cease treatment [58].

#### Neoplastic

**Fig. 12:**
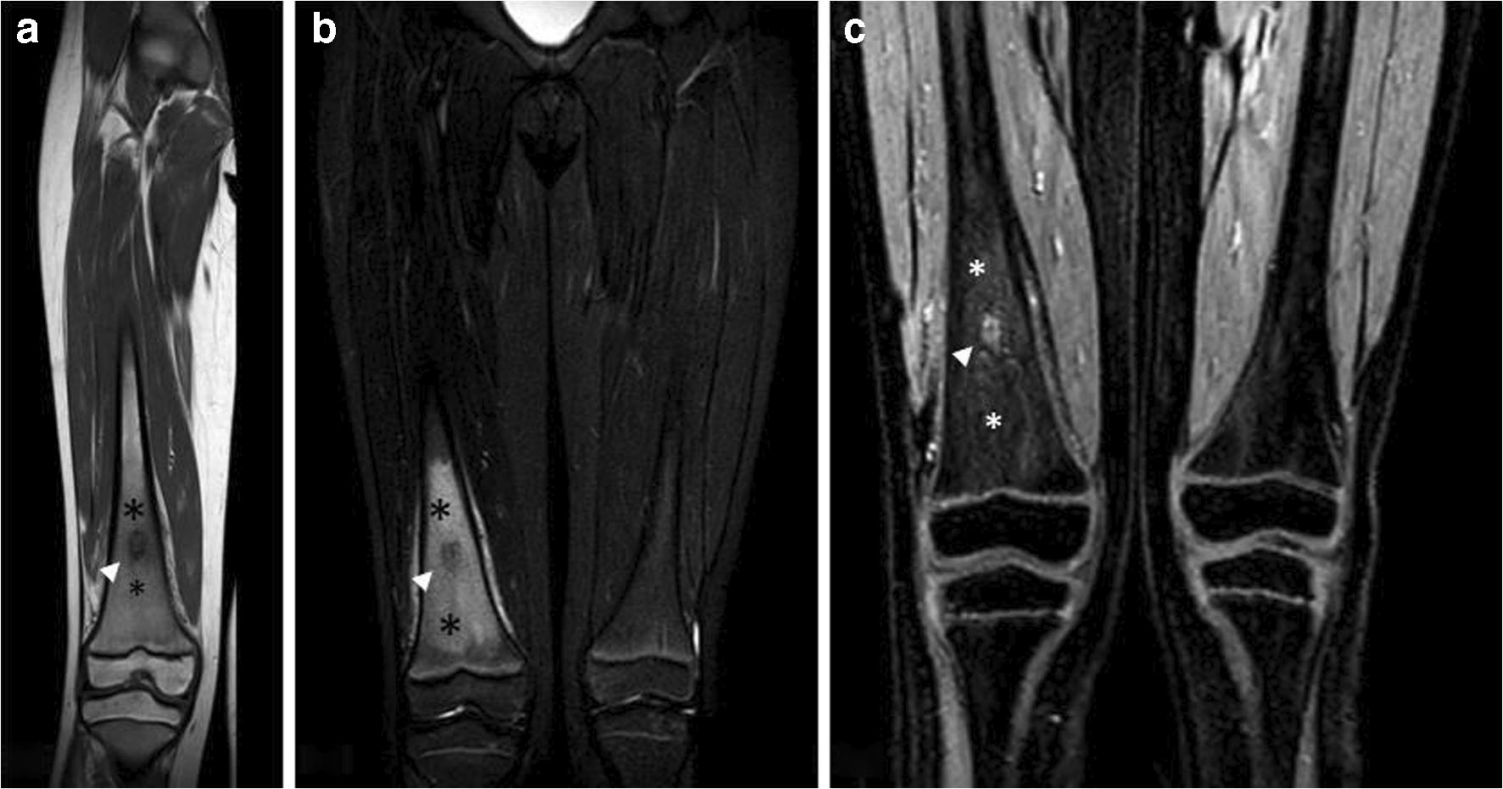
**a**, **b**, **c** Benign neoplastic (osteoid osteoma). A 8-year-old male patient with pain in the end of the left thigh worsening at night. T1w imaging (a) shows a hypointense lesion with well-defined margins (*arrowhead*) surrounded by a slightly hypointense halo (*). STIR imaging (b) demonstrates the edematous nature of the halo (*) and well highlights the great extension of the perilesional ELMSI itself. Contrast-enhanced T1w imaging (c) shows the enhancement of the nidus (*arrowhead*) and the slight enhancement of the perilesional marrow signal alterations (*)

**Fig. 13:**
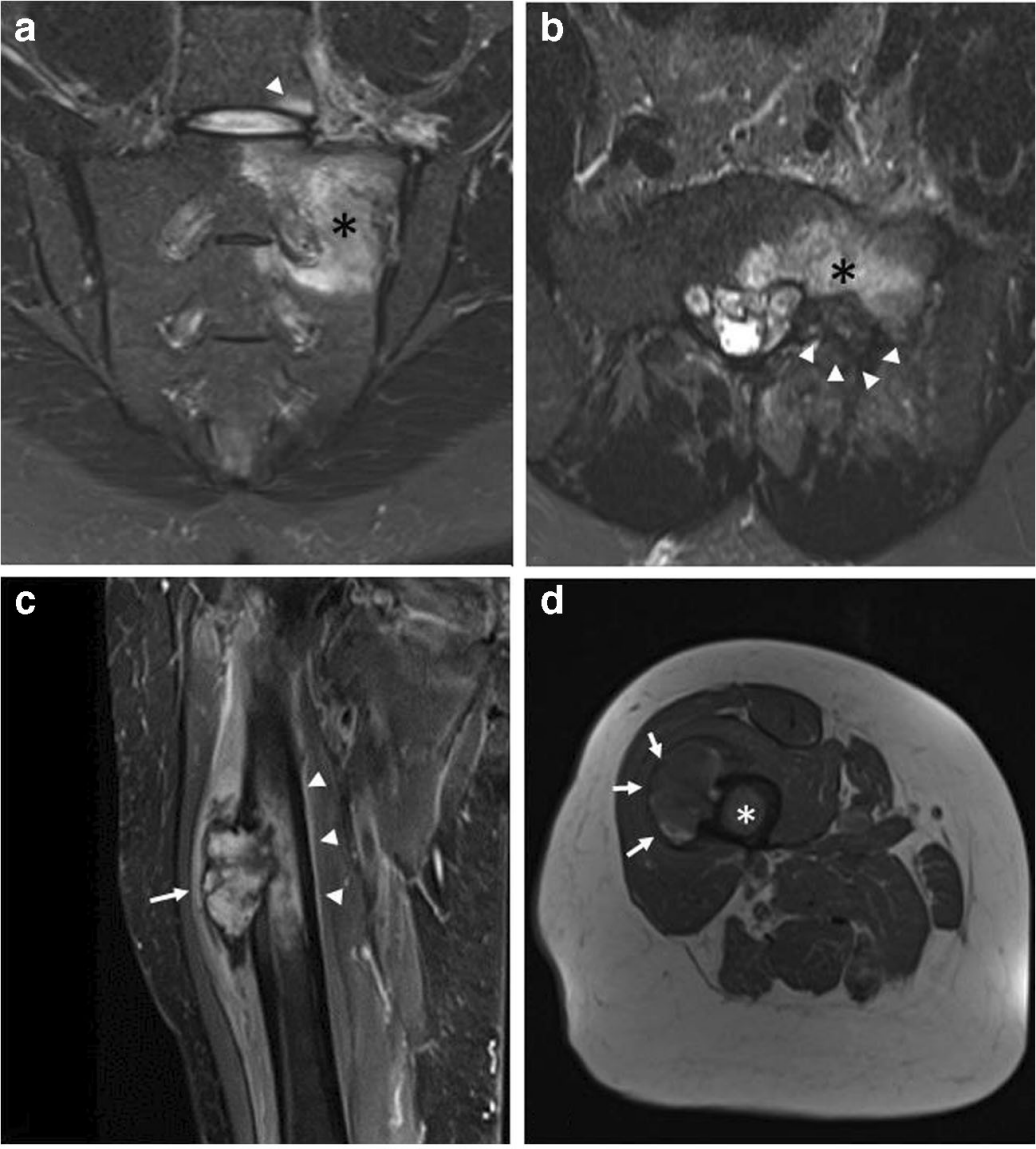
**a**, **b**, **c**, **d** Benign and malignant neoplastic. ELMSI from an osteoblastoma of the left hemisoma of S1 in a 36-year-old male patient (a–b). STIR coronal image (a) allows to detect an extensive ELMSI (*) involving the left hemisomas of S1 and S2 and the left posterior-lower corner of L5 (*arrowhead*). In STIR axial image (b) we can see both the ELMSI (*) and the benign nodule of S1 (*arrowheads*). Marrow alterations from a periosteal osteosarcoma of the proximal diaphysis of the right femur in a 74-year-old woman (c–d). STIR coronal image (c) shows the exophytic development of the tumor (arrow) and the extensive edemigenous reaction of the femoral spongiosa (*arrowheads*); in d, T1w image, on the axial plane, we can see the tumor (*arrows*) and the hypointense ELMSI (*) occupying the entire medullary canal

A great variety of benign and malignant neoplasms can determine ELMSI. Usually, benign lesions, such as osteoid osteoma (Fig. 12) and osteoblastoma (Fig. 13), show an important amount of surrounding marrow alterations [91]. Malignancy, represented by metastases and malignant primary bone tumors such as osteosarcoma (Fig. 13), Ewing’s sarcoma, and chondrosarcoma induce a minor quantity of ELMSI, except for chondroblastoma and Langerhans’s cell histiocytosis. The ELMSI associated with osteoid osteoma (present in more than 60% of patients) is probably caused by tumor-associated inflammatory mediators (including prostaglandin E2), while the one related to other tumors is thought to be caused by trabecular destruction and local inflammation [58]. Differentiating between the neoplastic mass and the ELMSI is crucial for the orthopedic surgeon and so, to do this task appropriately, radiologists can be helped by DwI and chemical shift–weighted imaging sequences [58].

#### Iatrogenic

**Fig. 14:**
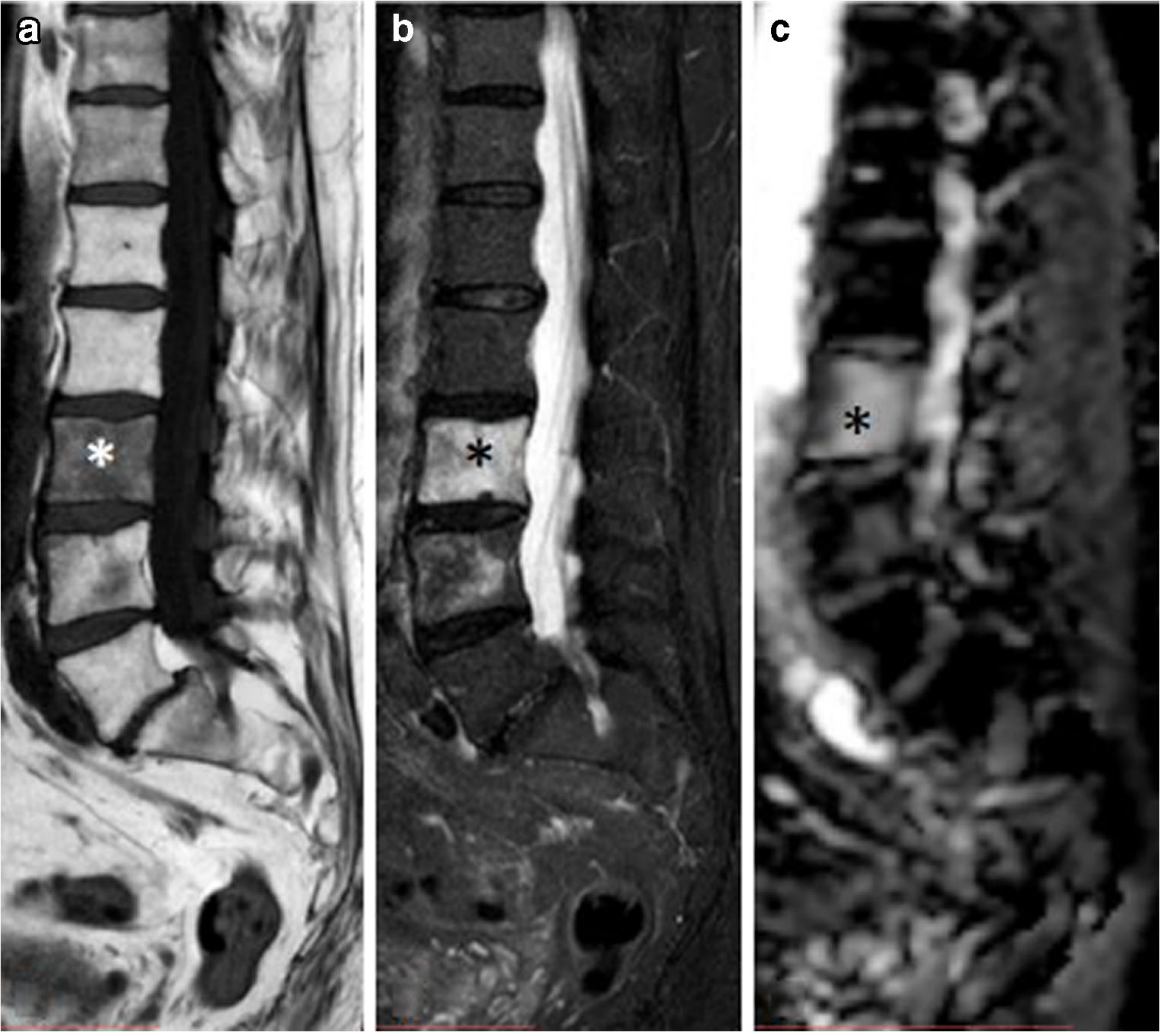
**a**, **b**, **c** Iatrogenic. A 73-year-old male patient with recent radiotherapy of the lumbar spine for the treatment of a non-Hodgkin lymphoma. T1w (a) and STIR (b) images demonstrate the presence of an abundant ELMSI (*) and the ADC map (c) shows the absence of diffusion restriction in L3, that was previously affected by the disease

It can derive from local surgery (Fig. 7), radiotherapy or the use of some drugs such as calcineurin inhibitors [58]. It occurs most frequently post radiotherapy and in this context it is thought to be a relatively rapid change that takes place acutely (1–14-day time frame) (Fig. 14). Even if some authors did not detect ELMSI beyond 21 days post-radiotherapy [92], its presence, intensity, and duration can vary significantly depending on the type and location of the treatment.

#### Metabolic

Irrespective of the presence of cortical erosion, ELMSI at the insertion site of the pathologic tendon is associated with the inflammatory response to microscopic deposits of hydroxyapatite within the bone. Another tendinopathy linked to the presence of abundant ELMSI is represented by calcium pyrophosphate deposition (CPPD), especially in the acute phase [93]. Less frequently, ELMSI can be caused by the presence of intraosseous tophi in patients with gout [58].

#### Neurological

Charcot’s joint is a progressive degenerative, inflammatory, and destructive joint disease with abnormal pain sensation and proprioception. ELMSI is often associated with this disorder, due to the polyneuropathy caused by diabetes mellitus; the most commonly joints affected are foot and ankle [94]. Even if conventional X-rays acquisitions are traditionally the standard imaging technique for diagnosing, staging, and monitoring the disease, MRI can be very sensitive to obtain an early diagnosis and determine the course of the healing process. In fact, ELMSI is an early sign of Charcot’s joint and until when a significant amount is seen on MRI; consequently, off-loading therapy (with removable total contact casts) must be continued. Only when a significant decrease or complete disappearance is detected, the cast can be removed, and an orthopedic shoe adapted [94, 95]. Differentiation between acute Charcot foot and infection (osteomyelitis) is not so simple because clinical exam and X-rays acquisitions may be not conclusive. However, differentiation is important because treatment varies depending on the disease present and MRI can be very helpful. In osteomyelitis, ELMSI tends to be extensive and usually involving a single bone, while it tends to be periarticular and subchondral in acute Charcot osteoarthropathy. Moreover, a focal involvement of the weight-bearing surfaces of the toes or the metatarsal heads, absence of deformity, presence of sinus tract, and soft tissue infection are suggestive of osteomyelitis, while an involvement of several joints/bones, deformity along with bone debris usually present with acute Charcot osteoarthropathy [96].

## Conclusions

The old term “bone marrow edema” is nowadays replaced by “ELMSI,” an acronym indicating the well-known MR T2w hyperintense marrow areas that can be caused not only by a simple real edema, but by various occurrences, such as lymphocytic infiltrates, early fibrosis, necrosis, hemorrhage, and aggregates of bone vessels. ELMSI is not always a pathological finding and may be observed, for example, in healthy children knee, where it is generally a transitory, self-limiting condition related to growth plate closure. ELMSI presents a dynamic behavior and its evolution can largely vary; MRI can track its changes over time, becoming a marker to follow the evolution and therapeutic response of the underlying disease. Unenhanced MR can easily detect ELMSI and help in clarifying its cause. However, particularly when the cause is unknown, it can be difficult to predict if an ELMSI area will completely resolve or progress towards osteonecrosis, and in order to solve this problem, perfusion studies are ongoing with promising preliminary results.

In conclusion, ELMSI proved to be a very interesting topic and deserves to be explored further.

## Abbreviations

AVN: Avascular necrosis
DwI: Diffusion-weighted imaging
ELMSI: Edema-like marrow signal intensity
RA: Rheumatoid arthritis
SIFK: Subchondral insufficiency fractures of the knee
STIR: Short T1 inversion recovery
